# New insights into the intestinal barrier through “gut-organ” axes and a glimpse of the microgravity’s effects on intestinal barrier

**DOI:** 10.3389/fphys.2024.1465649

**Published:** 2024-10-10

**Authors:** Hong-Yun Nie, Jun Ge, Guo-Xing Huang, Kai-Ge Liu, Yuan Yue, Hao Li, Hai-Guan Lin, Tao Zhang, Hong-Feng Yan, Bing-Xin Xu, Hong-Wei Sun, Jian-Wu Yang, Shao-Yan Si, Jin-Lian Zhou, Yan Cui

**Affiliations:** ^1^ Department of General Surgery, The Ninth Medical Center of PLA General Hospital, Beijing, China; ^2^ Clinical laboratory, The Ninth Medical Center of the PLA General Hospital, Beijing, China; ^3^ 306th Clinical College of PLA, The Fifth Clinical College, Anhui Medical University, Beijing, China; ^4^ Department of Disease Control and Prevention, The Ninth Medical Center of PLA General Hospital, Beijing, China; ^5^ Special Medical Laboratory Center, The Ninth Medical Center of PLA General Hospital, Beijing, China; ^6^ Department of Pathology, The Ninth Medical Center of PLA General Hospital, Beijing, China

**Keywords:** intestinal barrier damage, gut-organ axis, extraintestinal organs, signaling pathway, microgravity

## Abstract

Gut serves as the largest interface between humans and the environment, playing a crucial role in nutrient absorption and protection against harmful substances. The intestinal barrier acts as the initial defense mechanism against non-specific infections, with its integrity directly impacting the homeostasis and health of the human body. The primary factor attributed to the impairment of the intestinal barrier in previous studies has always centered on the gastrointestinal tract itself. In recent years, the concept of the “gut-organ” axis has gained significant popularity, revealing a profound interconnection between the gut and other organs. It speculates that disruption of these axes plays a crucial role in the pathogenesis and progression of intestinal barrier damage. The evaluation of intestinal barrier function and detection of enterogenic endotoxins can serve as “detecting agents” for identifying early functional alterations in the heart, kidney, and liver, thereby facilitating timely intervention in the disorders. Simultaneously, consolidating intestinal barrier integrity may also present a potential therapeutic approach to attenuate damage in other organs. Studies have demonstrated that diverse signaling pathways and their corresponding key molecules are extensively involved in the pathophysiological regulation of the intestinal barrier. Aberrant activation of these signaling pathways and dysregulated expression of key molecules play a pivotal role in the process of intestinal barrier impairment. Microgravity, being the predominant characteristic of space, can potentially exert a significant influence on diverse intestinal barriers. We will discuss the interaction between the “gut-organ” axes and intestinal barrier damage, further elucidate the signaling pathways underlying intestinal barrier damage, and summarize alterations in various components of the intestinal barrier under microgravity. This review aims to offer a novel perspective for comprehending the etiology and molecular mechanisms of intestinal barrier injury as well as the prevention and management of intestinal barrier injury under microgravity environment.

## Graphical Abstract

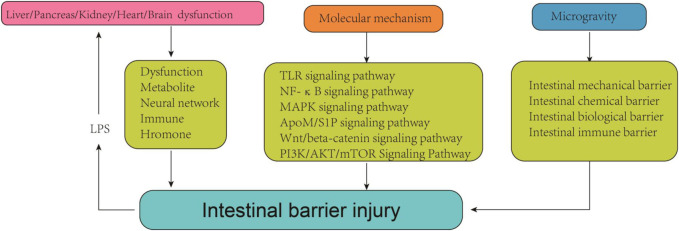

## 1 Introduction

Gut is the largest digestive and immune organ, as well as the principal interface between humans and the environment (Yang and Yu, 2021). In addition to its role in nutrient digestion and absorption, the gut also establishes a distinctive mucosal barrier that protect the host from harmful substances and pathogens (Hao et al., 2022). In general, intestinal barrier can be categorized into four components: the mechanical, immune, chemical, and biological barriers. Each component is supported by its corresponding structural foundation. An intact intestinal barrier plays a crucial role in upholding gastrointestinal function and overall human health (Di Sabatino et al., 2023). Once the intestinal barrier is damaged, it may further trigger strong inflammatory responses, autoimmune disorders, metabolic dysregulation, and deterioration of organic lesions (Albillos et al., 2020). Upon the critical importance of intestinal barrier integrity, extensive research has been conducted to investigate the causes and underlying mechanisms of intestinal barrier damage.

**FIGURE 1:**
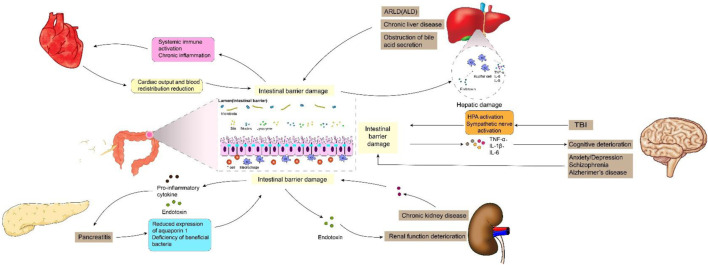
Intestinal barrier damage in the “gut-organ” axes.

Previous studies have shown that intestinal barrier damage is often associated with intestinal diseases such as intestinal infection, irritable bowel syndrome, ischemia-reperfusion (I/R) injury, inflammatory diseases and intestinal malignancies (Gao et al., 2023; Zhang et al., 2020). In recent years, the interconnection between the gastrointestinal tract and extraintestinal organs has gradually been elucidated. The concepts of “gut-liver axis,” “gut-kidney axis,” and “gut-heart axis” have been proposed, unequivocally indicating the intricate communication network between the intestines and other organs (Lombardi et al., 2024; Xiao et al., 2023). Studies have demonstrated that functional impairment of certain extraintestinal organs can induce oxidative stress, provoke an inflammatory response, and disrupt metabolic homeostasis in the intestine, thereby impacting intestinal barrier function. When the integrity of the intestinal barrier is compromised, it results in increased intestinal permeability and subsequent release of gut-derived endotoxin (Nie et al., 2023). Subsequently, these endotoxins can exert their effects on the target organs via the circulatory system, leading to further deterioration of the extraintestinal organs and eventually forming a vicious cycle of “gut-organ” axis disorder, which brings greater challenges to the management of intestinal barrier injury. It can be speculated that the disturbance of these axes plays a crucial role in the occurrence and progression of intestinal barrier damage (Figure 1). It is noteworthy that during the initial phase of functional impairment in certain organs, such as liver injury, kidney injury, and cardiovascular disease, increased intestinal permeability and endotoxin release can be detected, which helps to identify some high-risk adverse events early (Lee et al., 2018). At the same time, restoring intestinal barrier function could potentially serve as a therapeutic approach to mitigate extraintestinal organs injuries.

Signal transduction pathway, serving as the foundation for various cellular functions such as proliferation, differentiation, apoptosis, metabolism, oxidative stress, and immune response, facilitates the transmission of extracellular signaling molecules to the cell via cell membrane or intracellular receptors. Its proper functioning is indispensable for maintaining cellular and organ homeostasis (Chou et al., 2022). Dysregulation of cell signaling pathways has been demonstrated to be closely associated with the disruption and restoration of the intestinal barrier in previous studies (He et al., 2022). The elucidation of the molecular mechanism underlying intestinal barrier injuries holds significant importance in preventing such injuries and enhancing intestinal barrier homeostasis. Simultaneously, a comprehensive understanding of relevant mechanisms can aid in the development of targeted drugs to reverse ectopic signaling pathways, thereby offering potential therapeutic approaches for treating intestinal barrier injury.

The exploration of space by humanity is consistently accompanied by fervent enthusiasm. Aerospace medicine, as a prominent domain in contemporary medical research, also garners significant attention. Weightlessness, being an essential and inevitable factor within the space environment, exerts a profound and intricate influence on organisms (Bharindwal et al., 2023). Studies have demonstrated that microgravity or simulated microgravity (SMG) can induce alterations in the physiopathological state of the digestive system and exert diverse effects on various intestinal barriers, encompassing mechanical, immune, chemical, and biological barriers (Yang et al., 2020). Consequently, it is imperative to summarize the modifications occurring in the intestinal barrier under microgravity conditions and to uphold gastrointestinal homeostasis among astronauts in space.

## 2 The key roles of intestinal barrier damage in “gut-organ” axes cross talk

### 2.1 Gut–liver axis

The gut and liver are anatomically and physiologically closely interconnected, with this intimate association originating from a shared derivation from the ventral foregut endoderm during embryogenesis (Zorn and Wells, 2009). The gut-liver axis (GLA) is characterized by bidirectional interaction between the intestine and the liver. Bile acids and bilirubin synthesized by hepatocytes are released into the duodenum via the biliary tract, while nutrients from the intestinal lumen are transported to the liver through the portal circulation. The optimal functioning of GLA is crucial for efficient nutrient absorption and waste elimination. The liver damage, however, can lead to a decline in intestinal barrier function and the production of enterogenic endotoxins, thereby breaking the balance of GLA and exacerbating further deterioration of both the liver and intestine. The reported findings indicate a significant increase in the sensitivity of intestinal epithelial cell apoptosis and the nitration of intestinal tight junction (TJ) and adhesive junction (AJ) proteins in mice with alcohol-related liver disease (ARLD) (Rungratanawanich et al., 2023). Xiao et al. (2020) reported a decrease in the expression of ZO-1, Claudin-1, Claudin-4, and Reg3g in the intestines of mice with liver injury, leading to an increase in intestinal permeability. Furthermore, there was a negative correlation observed between the expression levels of these proteins and serum endotoxin levels (Assimakopoulos et al., 2012). Hypoxia-inducible factor 1a (HIF-1a) plays a crucial role in the transcriptional regulation of intestinal barrier integrity and inflammation, governing the expression of various genes involved in barrier protection, including intestinal trefoil factor (ITF), CD73, p-glycoprotein (P-gp), cathelicidin, claudin-1, and MUC3 (Fan et al., 2015; Saeedi et al., 2015). Shao et al. (2018) reported a significant reduction of hif-1a in the intestines of mice afflicted with alcoholic liver disease (ALD), leading to compromised intestinal barrier function and gut leakiness. Subsequent investigations demonstrated that upregulating hif-1a could serve as a potential therapeutic approach for ALD (Shao et al., 2018). In addition, the intestinal biological barrier is also affected by liver disease. Jiang et al. (2020) reported that liver injury can result in an increase of the relative abundance of potentially pathogenic *Escherichia* and *Staphylococcus*, as well as a reduction in the presence of SCFA-producing bacteria, such as *Prevotella, Faecalibacterium*, and *Clostridium*. Additionally, the presence of bacterial overgrowth in the small intestine and impaired intestinal motility have been observed in patients with chronic liver disease, leading to enterogenic endotoxemia (Voulgaris et al., 2021). The obstruction of bile acid secretion in patients with liver disease also plays a crucial role in the development of enterogenic endotoxemia. Bile acids exert direct antibacterial effects by inducing farnesol X receptor-mediated antimicrobial peptides such as angiopoietin 1, which effectively prevent bacterial overgrowth and enhance the integrity of intestinal epithelium. The bile acids and their salts, on the other hand, can also degrade endotoxin molecules into non-toxic subunits or polymerize them into colloidal molecules, thereby exhibiting antibacterial properties and regulating intestinal pH. In patients with liver disease, the impaired bile acid secretion and excretion compromise its ability to inhibit bacteria and regulate pH levels, as well as reduce the clearance of intestinal endotoxins. Moreover, elevated bile acid levels hinder the uptake and clearance of endotoxins by Kupffer cells (de Faria Ghetti et al., 2018).

The impairment of the intestinal barrier can also lead to hepatic damage via endotoxemia. The endotoxin, upon reaching the liver, binds to TLR4 and co-receptors CD14 and MD-2 in Kupffer cells, thereby initiating downstream signaling pathways that result in excessive production of pro-inflammatory cytokines such as TNF-α, IL-6, and IL-8 (Cho et al., 2018). The mice were initially engineered by Shao et al. (2018a) to develop intestinal barrier injury through targeted knockout of intestinal epithelial HIF-1a. Subsequently, they observed a significant increase in serum alanine aminotransferase and lipopolysaccharide levels, along with the presence of liver steatosis. The biologically active lysophospholipid sphingosine 1-phosphate (S1P) plays a crucial role in various biological processes, including cell adhesion, regulation of barrier function, proliferation, differentiation, migration, and protection of the intestinal barrier (Li et al., 2023a). Chen et al. (2023) discovered that elevating the level of S1P can effectively restore intestinal barrier function, reduce lipopolysaccharide (LPS) levels in plasma and liver, mitigate inflammation and apoptosis, as well as enhance liver function. Similarly, the findings of Wu et al. (2023) also demonstrated that upregulation of rat intestinal epithelial tight junction protein expression and modulation of rat intestinal microbiota imbalance can significantly attenuate levels of endotoxin and inflammatory cytokines in rats, while inhibiting the TLR4/NF-κB signaling pathway, thereby ameliorating hepatic pathological damage and oxidative stress. In conclusion, the normalization of GLA has garnered increasing attention through repairing intestinal barrier damage and reducing enterogenic endotoxin production. The levels of serum endotoxin in the blood have also been demonstrated to exhibit a positive correlation with the severity of advanced liver diseases, such as fibrosis and cirrhosis (Rungratanawanich et al., 2023).

### 2.2 Gut-pancreas axis

The gut and pancreas are intricately interconnected both anatomically and functionally (Floyel et al., 2023; Svegliati-Baroni et al., 2020). Previous studies have consistently demonstrated a strong association between intestinal barrier damage and pancreatic diseases. Epithelial cells (ECs) play a crucial role in the mechanical barrier of the intestine and are indispensable for maintaining its integrity (Benede-Ubieto et al., 2024). However, pancreatitis or local pancreatic injury can induce the release of a plethora of inflammatory factors such as TNF-α and IL-1β, thereby triggering activation of the caspase-3 pathway and subsequent apoptosis of intestinal mucosal epithelial cells (Chen et al., 2018). In addition, the expression of intestinal tight junction proteins, such as occludin and ZO-1, significantly decreases in acute necrotizing pancreatitis (ANP), leading to a significant increase in intestinal permeability. This phenomenon is closely associated with high-mobility group box-1 (HMGB1) (Huang et al., 2019). Lu et al.'s study demonstrated that pancreatic tissue damage exacerbates small intestinal capillary endothelial barrier dysfunction, which may be attributed to reduced expression of aquaporin 1 (AQP1) in the small intestine (Lu et al., 2017). The integrity of the intestinal biological barrier, which is established through the close adhesion of symbiotic bacteria to the intestinal epithelial mucosa, is compromised during acute pancreatitis (Li et al., 2020). Li et al. (2023) discovered alterations in the diversity of intestinal microbiota and a deficiency of beneficial bacteria in patients with pancreatitis. Specifically, a reduction in *Bacteroides* uniformis abundance among these patients resulted in decreased taurine levels and increased release of IL-17 within the intestine. This subsequently triggered neutrophil extracellular trap (NET) formation, exacerbating pancreatic injury. The abundance of probiotics, such as Blautia, exhibited a inverse association with the severity of acute pancreatitis (Zhu et al., 2019). Conversely, the presence of detrimental bacteria, including *Escherichia coli* and *Shigella*, amplified the NF-κB-mediated inflammatory response while concurrently suppressing protein expression (e.g., MUC2), thereby compromising intestinal barrier integrity (Zheng et al., 2019).

Following an increase in intestinal permeability, enterogenic endotoxins and inflammatory mediators can be transported to the pancreas *via* systemic circulation and mesenteric lymphatic pathways, ultimately exacerbating acute pancreatic inflammation. The results of pancreatic studies have demonstrated that promoting apoptosis in intestinal inflammatory cells to inhibit the activation of the NF-κB signaling pathway, reducing endotoxin secretion, decreasing phosphorylated-p65 (p-p65) expression, and increasing IκBα expression can effectively enhance the pathological outcome of both pancreatic and intestinal tissues (Pan et al., 2024). Similarly, Mei et al. (2021) discovered that the mitigation of pro-inflammatory cytokine production (TNF-α, IL-1β, CXCL2 and MCP1) and endotoxin in the ileum, along with activation of the Nrf2/HO-1 pathway, could effectively restore ileum injury and barrier dysfunction associated with severe pancreatitis. Furthermore, there was a significant reduction observed in serum amylase levels, lipase levels, and pancreatic pulp peroxidase activity. The team led by Li et al. demonstrated enhanced intestinal barrier function and reduced intestinal inflammation in mice through microbiota transplantation (MT) and NLRP3 knockout. Subsequently, they observed a decrease in pancreatic neutrophil infiltration and necrosis (Li et al., 2020). In fact, there are limited therapeutic interventions for intestinal damage associated with acute pancreatitis, and the majority of treatments focus on hormone-mediated inhibition of pancreatic enzyme secretion, which does not effectively address intestinal injury. However, during the early stages of acute pancreatitis, enterogenic endotoxins and inflammation can propagate to the pancreas and other organs through the gut-pancreas axis, leading to severe consequences (Pasari et al., 2019). Therefore, it is imperative to address intestinal injury in association with AP by conducting comprehensive research on the gut-pancreas axis.

### 2.3 Gut-kidney axis

The correlation between the gut and renal is gradually being elucidated. Simultaneously, multiple studies have demonstrated the potential involvement of kidneys in the progression of intestinal barrier impairment. Clinical trials have demonstrated that patients with stage IIIb-IV chronic kidney disease (CKD) exhibit the accumulation of enterogenous uremic toxins (UT), such as indoxyl sulfate (IS) and p-toluene sulfate (PCS), along with increased intestinal permeability and constipation (Cosola et al., 2021). The presence of chronic kidney disease (CKD) has been associated with intestinal villi shortening, elongation of crypts, and infiltration of the lamina propria (Georgopoulou et al., 2024). In addition, serum levels of endotoxin, IL-6, IL-8, and IL-10 were significantly elevated in patients with stage I-IV CKD, while intestinal occludin and claudin-1 exhibited significant reduction in expression. Furthermore, their expression showed a negative correlation with systemic endotoxemia (Georgopoulou et al., 2024). The impairment of the intestinal barrier is partially facilitated by urea (Lau et al., 2015). With an increase in intestinal barrier permeability, endotoxins, bacteria, and toxins are able to enter the circulatory system, leading to further deterioration of renal function. Additionally, kidney dysfunction can also impact the gut microbiome by causing a reduction in the population of proteolytic bacteria, altering the ratio of aerobic and anaerobic bacteria, and compromising intestinal epithelial barrier integrity. Simultaneously, changes in the microbiome can lead to the generation of potentially toxic compounds that are typically eliminated by renal excretion (Mertowska et al., 2021; Jaglin et al., 2018; Alexeev et al., 2018). Microorganisms such as *Enterobacterium*, *Enterococcus*, *Bifidobacterium*, and *Bacteroides*, which are responsible for the production of short-chain fatty acids, were found to exhibit reduced abundance in blood and fecal samples from patients with CKD (50). Furthermore, there was a negative correlation observed between the concentration of short-chain fatty acids in the bloodstream and the severity of renal insufficiency (Wang et al., 2019).

The discovery of the gut-kidney axis has also established that disruption of intestinal homeostasis can promote the onset and progression of renal disease. Several recent studies have initiated the development of therapies for renal dysfunction by focusing on restoring intestinal barrier function and microbial balance. The study conducted by Yang et al. (2024) demonstrated that the renal toxicity induced by zinc could be mitigated through rectifying colon injury, restoring ZO-1 protein expression, and reestablishing the structure of intestinal flora. Wang et al. (2024) demonstrated that oral administration of Lactococcus cremoris D2022 can enhance the production of short-chain fatty acids (SCFA) in cecum samples, ameliorate intestinal barrier function, and ultimately mitigate kidney inflammation. Additionally, in the study conducted by Yang et al. (2024), it was observed that oral administration of the probiotic *Lactobacillus* reuteri exhibited significant enhancement in intestinal barrier function impairment associated with AKI, while also regulating the composition of intestinal microbiota and its related metabolites. Consequently, there was a reduction in serum creatinine and urea nitrogen concentrations, along with protection against renal cell necrosis and apoptosis (Yang et al., 2024). The findings suggest that the preservation of gut barrier integrity and regulation of gut microbiota and associated metabolites should not be overlooked in the treatment of kidney disease. The significance of the kidney in maintaining proper physiological functioning of the human body goes without saying (Li et al., 2024); however, it remains one of the most overlooked organs. This can be attributed not only to insufficient knowledge and action in the field of prevention but also to the fact that most kidney diseases are asymptomatic during their initial stages. Early detection and diagnosis of kidney disease are severely limited due to a lack of sensitive and specific molecular markers indicating the progression towards a particular disease entity. Therefore, it is crucial to explore novel disease markers for predicting and tailoring personalized treatments for kidney diseases. Evaluation of gastrointestinal function through comprehensive analysis of gut microbiota composition holds potential for enhancing diagnostic capabilities, necessitating further investigation into the enterorenal axis.

### 2.4 Gut-heart axis

The concept of the gut-heart axis elucidates the intricate connection between intestinal pathology, the intestinal microbiome, and cardiovascular disease. Disorders of the gut-heart axis are characterized by alterations in intestinal permeability and disruption of the microbiome. Then, the gastrointestinal microbiota or its derivatives traverse the intestinal epithelial barrier in a non-physiological manner, leading to systemic immune activation and chronic inflammation, which subsequently impact cardiovascular function (Witkowski et al., 2020). Additionally, the contribution of cardiovascular disease to intestinal barrier damage is significant. In patients with heart failure, reduced cardiac output and blood redistribution result in decreased intestinal perfusion, leading to ischemia and hypoxia in the mucous membrane of the villous structure of the intestinal wall. This alteration in the intestines increases permeability of the intestinal wall, causing disturbances in fluid metabolism, disruptions in intestinal microbial balance, translocation of bacteria from the intestines into the circulatory system, and a increase in endotoxins, thereby promoting a characteristic inflammatory state (Xu et al., 2024). The lactulose/mannitol test revealed a 35% elevation in small intestine permeability, while the sucralose test demonstrated a significant increase of 210% in large intestine permeability among patients with chronic heart failure (CHF) (Sandek et al., 2007). The disruption of intestinal barrier integrity and the downregulation of tight junction protein expression have been reported to be associated with neutrophil extracellular trap (NET) (Wang et al., 2018). Hoel et al. (2020) discovered that COVID-19 patients with cardiac involvement exhibited elevated levels of intestinal leakage markers (LPS-binding protein, LBP) and intestinal epithelial cell damage markers (intestinal fatty acid-binding protein, IFABP), indicating the potential existence of a Gut-heart axis in COVID-19. Recently, Blöbaum et al. (2023) conducted a groundbreaking study where they successfully diagnosed intestinal barrier dysfunction in patients with atrial fibrillation (AF). Their research findings revealed an elevation in circulating biomarkers associated with intestinal mucosal inflammation, such as mucosal adhesion molecule MAdCAM-1, and indicators of intestinal epithelial damage like intestinal fatty acid binding protein (IFABP) present in plasma among individuals experiencing early stages of atrial fibrillation (AF). Additionally, surrogate plasma markers indicating increased intestinal permeability were also detected, including LPS, CD14, and LPS-binding proteins. Moreover, Du et al. (2020) observed alterations in the composition of intestinal microbiota (particularly *firmicutes* and *Bacteroidetes*) as well as significant elevations in metabolites associated with the microbiome, including short/medium chain fatty acids, arginine, and tryptophan derivatives, in rats exhibiting cardiac hypertrophy. These changes are implicated in the impairment of intestinal barrier integrity induced by heart failure.

In recent years, the restoration of intestinal barrier integrity has emerged as a novel approach for the treatment of heart dysfunction by researchers. The correlation between intestinal permeability and markers of heart injury was found to be positive (Wu et al., 2024). Cui et al. (2023) demonstrated that enhancing the expression of tight junction proteins (ZO-1, occludin) and reducing intestinal permeability and inflammation can lead to improved cardiac function, as well as decreased serum CK-MB and LDH expression. The microbiota, as a crucial component of the intestinal biological barrier, is increasingly being targeted for heart failure treatment (Violi et al., 2023; Bianchi et al., 2022). Regulating gut microbiota through dietary interventions, probiotics administration, antibiotic therapy, fecal transplants, and microbial enzyme inhibitors can effectively enhance cardiac function and reduce mortality associated with heart failure. These interventions involve strategies such as optimizing the firmicutes/Bacteroidetes ratios and promoting the growth of beneficial microbiota (bacteroidetes and heterobacteroidetes) (Jia et al., 2019). The recognition of intestinal barrier damage as a risk factor for cardiovascular disease is increasingly growing. In certain cases of early heart disease, gut-derived low-grade endotoxemia may be present (Violi et al., 2023). Therefore, it is imperative to conduct early assessment of intestinal permeability and detection of endotoxins in high-risk groups for cardiovascular disease to facilitate timely intervention.

### 2.5 Gut-brain axis

The bidirectional communication between the brain and gut occurs *via* systemic immune pathways, neural networks, endocrine hormones, and microbiota axes (Morais et al., 2021). Studies have demonstrated that traumatic brain injury (TBI) exerts detrimental effects on the gastrointestinal tract through hormonal regulation. TBI triggers activation of the hypothalamic-pituitary-adrenal (HPA) axis, leading to an elevation in cortisol levels (Li et al., 2023). This surge in cortisol enhances intestinal barrier permeability, ultimately resulting in intestinal leakage (Morais et al., 2021). Consequently, enteric pathogens infiltrate the bloodstream and induce systemic inflammatory response syndrome, thereby releasing a plethora of cytokines and chemical mediators into the systemic circulation. The sharp escalation of pro-inflammatory cytokines such as TNF-α, IL-1β, and IL-6 accelerates cognitive function deterioration (Milleville et al., 2021). In addition, gut can be influenced by brain injury through neuroregulation. TBI can induce sympathetic overactivity, leading to an elevation in circulating catecholamine levels, subsequently resulting in reduced blood flow within the gastrointestinal tract and causing gut dysmotility as well as damage to the intestinal barrier (Montgomery et al., 2016). There is also a correlation between anxiety and intestinal disorders. A clinical study investigation revealed that patients with depression/anxiety disorder exhibited dysregulation of the gut microbiota and significantly elevated levels of LPS, zonulin, and FABP2 in comparison to healthy subjects. Assessing markers of intestinal permeability can aid in interventions for depression and anxiety (Stevens et al., 2018). Furthermore, studies have demonstrated increased intestinal permeability in individuals with schizophrenia, characterized by tight junctions disruption, adhesive junctions dysfunction, and heightened bacterial translocations (Maes et al., 2019). The assessment of the lactulose to mannitol ratio revealed a significantly higher index in patients with schizophrenia compared to the control group (Ishida et al., 2022). There is a strong correlation between gut flora and the central nervous system. The microbiota has the ability to regulate the synthesis of neurotransmitters that impact the gut-brain axis, such as tryptophan. Tryptophan serves as a crucial precursor for aminergic neurotransmitters and cannot be synthesized by the human body; however, it is produced by gut microbes through the shikimate pathway. Notably, Alzheimer’s disease onset is associated with tryptophan secretion in intestinal flora. Although the gut and brain are physiologically and anatomically distant, neuroimmune links between the two are gradually being discovered, which could help in the development of more intestinal biomarkers to make the prevention and diagnosis of neurological diseases more effective.

### 2.6 Gut-liver-brain axis

Gut-liver-brain axis serves as a tripartite communication pathway, garnering increasing attention due to its paramount significance (Muhammad et al., 2022). Maintenance of homeostasis within the gut-hepato-brain axis is imperative for optimal brain functionality, contingent upon the intestinal barrier’s integrity and hepatic filtration efficacy. Intestinal tract is the primary reservoir of bacteria within the human body, and its barrier function effectively prevents the translocation of intestinal toxins into capillaries. The liver functions as a prominent “detoxification factory,” efficiently metabolizing and filtering exogenous and endogenous metabolites, bacterial products, and toxins (Wiest et al., 2017). Impairment of either the liver or intestinal barrier can lead to an exacerbation of abnormal gut-liver-brain axis states. Specifically, the impairment of the intestinal barrier facilitates the translocation of bacteria or their metabolites into the liver, thereby precipitating various hepatic disorders (Bauer et al., 2022). In addition, the gut can also affect nerve signals between the gut and the brain by influencing the production of various peptides or hormones. Subsequently, the brain innervates liver and intestinal activity through neuroimmunomodulation, exacerbating the disorder of the enteroliver-brain axis (Socala et al., 2021). Studies have demonstrated that alterations in the composition of intestinal microbiota and its metabolites, along with increased intestinal permeability, play a pivotal role in the pathogenesis of hepatic encephalopathy (Aguirre Valadez et al., 2016). In individuals with liver dysfunction, compromised integrity of the intestinal barrier can further exacerbate hepatic failure. In patients with cirrhosis, impaired liver function hampers ammonia metabolism, leading to elevated blood ammonia levels. Subsequently, ammonia traverses the blood-brain barrier and accumulates within the brain parenchyma, thereby triggering hepatic encephalopathy and cognitive impairment (Milosevic et al., 2019). Hyperammonemia can induce microglial activation, indicating the generation of inflammation in the central nervous system (Aitbaev et al., 2017). Furthermore, the gut microbiota also plays a pivotal role in hepatic encephalopathy development. Research has demonstrated that alterations in intestinal motility and decreased bile acid levels among cirrhosis patients contribute to an overgrowth of intestinal bacteria, resulting in dysbiosis of the gut flora. Bajaj et al. demonstrated that, in comparison to the control group, patients with hepatic encephalopathy exhibited an elevated proportion of enterobacteriaceae, Fusobacteriaceae, and Veillonaceae in their intestinal mucosal flora. Furthermore, these microbial groups were positively correlated with intestinal inflammation. Additionally, enterobacteriaceae was found to be associated with astrocyte changes linked to hyperammonemia (Ahluwalia et al., 2016). These findings suggest a potential association between specific gut microbiota and cirrhosis-related brain dysfunction.

## 3 Causes of intestinal barrier damage

The occurrence of intestinal inflammation is a pathological process triggered by external stimuli or pathogen invasion in the intestinal tissues (Medzhitov, 2010). In the early stages of inflammatory diseases, such as acute intestinal infections, pro-inflammatory cytokines (IL-1β, IL-6, and TNF-α) are released in large quantities and cause an inflammatory cascade. Conversely, anti-inflammatory cytokines (IL-4, IL-10, and IL-13) can suppress the inflammatory response and mitigate damage caused by excessive inflammation. The dysregulation of inflammatory/anti-inflammatory factors results in the infiltration of immune cells, concurrently activating signaling targets such as NF-κB and MAPK, thereby influencing the expression of downstream proteins (intestinal barrier proteins, immune receptors, and related enzymes) (Dong et al., 2019), ultimately leading to an elevation in intestinal permeability (Wells et al., 2017). In addition, intestinal infection can induce apoptosis of intestinal epithelial cells, resulting in the subsequent release of damage-associated molecular patterns. This leads to a persistent state of heightened inflammation within the intestinal mucosa and impairs the functionality of goblet cells and mucosal epithelial regeneration, thereby compromising the repair process of the damaged intestinal barrier (Jiang et al., 2010). However, the presence of ulcers, bleeding, and an imbalance in the mucosal environment can further compromise the integrity of the intestinal mucosal barrier, ultimately resulting in a detrimental cycle of ongoing mucosal damage and destruction, impaired reconstruction capacity, and exacerbated homeostatic imbalance (Chang et al., 2013).

Studies have demonstrated that cigarette smoking exerts both direct and indirect effects on the gastrointestinal tract, encompassing oxidative damage, compromised immune cell functionality, alterations in epigenetic patterns, as well as changes in microbial composition that collectively contribute to intestinal barrier dysfunction (Papoutsopoulou et al., 2020). The study conducted by Berkowitz et al. (2019) demonstrated that smoking adversely affects the integrity of the intestinal barrier in the small intestine through its impact on Pan’s cells, specialized epithelial cells located in this region responsible for secreting antimicrobial peptides crucial for regulating microbial growth. When mice were exposed to cigarette smoke condensate (CSC), specific alterations in Paneth cell granules in the ileum were observed, leading to a decrease in antimicrobial peptide production and bactericidal capacity. Furthermore, CSC induced an imbalance in the gut bacterial community and heightened susceptibility to bacterial infection-induced ileal damage in mice. The potential risk of intestinal injury due to chronic smoke exposure may be attributed to modifications in mucin distribution within the intestinal epithelium and alterations in flora composition (Allais et al., 2016).

In addition, other irritants including alcohol, radiation, and drug abuse are important triggers for intestinal barrier damage. The activation of pro-oxidases/genes and the inhibition of antioxidant levels, including glutathione, have been reported as key factors contributing to alcohol-induced intestinal damage, leading to heightened oxidative and nitrification (nitrosation) stress. Similarly, factors such as physical stimulation (radiation) and bacterial infection can also impact intestinal barrier damage by inducing imbalances in intestinal oxidative stress. When there is an excessive oxidation of intestinal tissues, it can hinder the regeneration of intestinal epithelial cells (IECs), increase the disruption of tight junction integrity, and reduce the secretion of antioxidants and other physiological processes that affect intestinal barrier function (Gao et al., 2023). Oxidative stress has the ability to directly impair IECs and activate various pathways related to oxidative and anti-oxidative stress, thereby regulating the extent of intestinal damage.

The occurrence of intestinal barrier damage is often not limited to a single cause. In addition to complex infection, inflammation, oxidative stress, apoptosis, and other pathological processes, intestinal ischemia/hypoxia and genetic factors are also closely associated with intestinal barrier damage, involving intricate mechanisms of interaction among them. To achieve a more accurate understanding of the etiology of intestinal injury, comprehensive and in-depth research on the molecular mechanism and injury mechanism needs to be conducted.

## 4 Molecular mechanism of intestinal barrier damage

### 4.1 TLR signaling pathway

**FIGURE 2:**
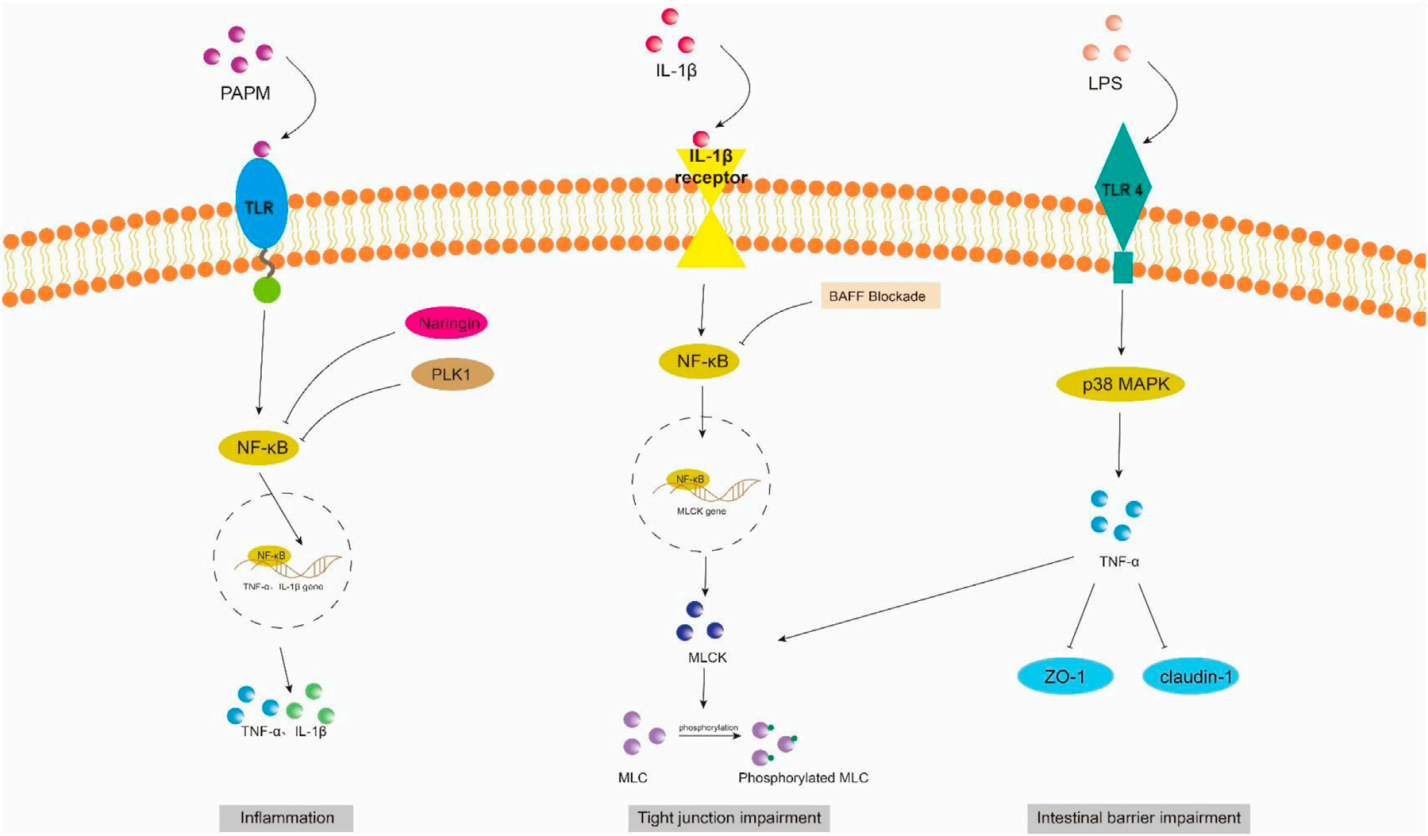
NF-κB and MAPK signaling pathway in intestinal barrier damage. PAPM, pathogen-associated molecular pattern; LPS, Lipopolysaccharides; TLR, Toll-like receptors; PLK1, Polo-like Kinase 1; ZO-1, zonula occluden-1.

Toll-like receptor (TLR) is an innate immune pattern recognition receptor, promptly triggering intracellular signaling cascades upon detection of pathogen-associated molecular patterns (PAMPs), including proteins, nucleic acids, and lipids derived from invading pathogenic microorganisms. Ultimately, this activation leads to the initiation of both non-specific and specific immune responses aimed at eliminating pathogens (Liu et al., 2024). LPS/TLR4 signaling pathway can be classified into MyD88-dependent and MyD88-independent pathways, both of which activate the NF-κB and MAPK pathways that regulate inflammation (Izadparast et al., 2022). In intestinal inflammatory diseases, the activation of the LPS/TLR signaling pathway induces a robust inflammatory response, resulting in increased apoptosis of intestinal epithelial cells (IECs) and downregulation of tight junction protein expression, ultimately leading to impaired intestinal barrier function (Figure 2). Recent studies have demonstrated that neutrophil extracellular traps (NETs) induce the activation of Toll-like receptor 9 (TLR9) signaling pathway in septic patients, thereby triggering endoplasmic reticulum (ER) stress in intestinal epithelial cells (IECs), leading to increased intestinal inflammation, apoptosis of IECs, tight junction injury, and ultimately compromising the mechanical barrier of the intestinal mucosal epithelium. Inhibition of TLR9-ER stress signaling can significantly ameliorate NETs-induced apoptosis of IECs and improve intestinal function (Sun et al., 2021). As custodians of small intestine crypts, stem cell-derived Panes cells not only enhance the function of stem cells and promote epithelial regeneration but also secrete highly potent antimicrobial peptides such as alpha-defensin and lysin.

However, in sepsis mice, it has been observed that the activation of the TLR4/ATF/CHOP signaling pathway by ER stress can induce apoptosis or dysfunction in Panzer cells, leading to exhaustion and subsequently impairing intestinal stem cell mobility, reducing secretion of antimicrobial peptides, exacerbating intestinal injury, and ultimately increasing mortality (Wang et al., 2022). Scholars have also discovered that the overexpression of pentosan-3 (PTX3) can inhibit the TLR signaling pathway, thereby reducing levels of inflammatory factors such as TNF-α, IL-1β, and interferon (INF)-γ. This inhibition helps alleviate apoptosis in intestinal epithelial cells (IECs) and promotes the expression of tight junction proteins ZO-1 and occludin between these cells, ultimately leading to a reduction in damage to the intestinal epithelium (Li et al., 2022).

#### 4.1.1 NF-κB signaling pathway

NF-κB is a crucial nuclear transcription factor involved in inflammation and immune response, while also regulating apoptosis and stress response. In sepsis, the activation of Transforming growth factor kinase 1 (TAK1) can be mediated by inflammatory factors such as TNF-α and IL-6. Upon activation, TAK1 phosphorylates the downstream inhibitory protein IκBα, leading to dissociation of NF-κBp65 from IκBα and subsequent translocation into the nucleus for transcriptional regulation of related genes. It has been observed that Polo-like kinase 1 (PLK 1) is suppressed in septic rats, leading to reduced expression of IκB-α and enhanced nuclear translocation of NF-κB p65. The combined downregulation of PLK1 and activation of NF-κB result in apoptosis of intestinal epithelial cells, thereby compromising the integrity of the intestinal mechanical mucosal barrier (Cao et al., 2020; Cao et al., 2018). At the same time, the expression levels of pro-caspase-3 and IκB-a were significantly upregulated upon pretreatment of human colon cells with NF-κB activity-inhibiting drugs, suggesting that inhibition of NF-κB can reduce the apoptosis of intestinal epithelial cells (Cao et al., 2020). Additionally, NF-κB signaling pathway can impair the integrity of the intestinal barrier by influencing the junctions between intestinal epithelial cells. The activated NF-κB p65 interacts with the promoter region of myosin light chain kinase (MLCK) and regulates the transcription of MLCK (Al-Sadi et al., 2008). Consequently, phosphorylation of MLC by MLCK triggers actin-myosin filament contraction, leading to downregulation of tight junction protein expression and increased intestinal permeability (Gatica-Andrades et al., 2017). Quan et al. (2020) discovered that rats treated with LPS exhibited significant increases in the phosphorylation levels of NF-κB p65 and IκBa, as well as MLCK and MLC, leading to a decrease in ZO-1 and occludin expression which compromised intestinal epithelial cell integrity and increased permeability. However, B-cell activator (BAFF) can inhibit the NF-κB/MLCK/MLC signaling pathway while increasing ZO-1 and occludin expression. Furthermore, a study conducted on animals demonstrated that the administration of gadolinium chloride (GdCl3) to sepsis rats resulted in a reduction of both systemic and intestinal inflammatory responses. This was primarily achieved through inhibition of NF-κB activation, leading to decreased MLCK expression, while promoting the expression of atrexin and ZO-1 (Zhao et al., 2021). Therefore, the restoration of the intestinal TJ barrier in endotoxemia can potentially be achieved by employing chemical compounds that possess inhibitory effects on NFκB and MLCK.

#### 4.1.2 MAPK signaling pathway

Mitogen-activated protein kinase (MAPK) is a cytoplasmic serine/threonine protein kinase that plays a crucial role in various physiological and pathological processes, including cell proliferation, differentiation, apoptosis, and survival. MAPK signaling network comprises three distinct pathways: the p38 mitogen-activated protein kinase (p38MAPK) pathway, the extracellular signal-regulated protein kinase 1/2 (ERK 1/2) pathway, and the c-Jun amino-terminal kinase (JNK/SAPK) pathway (Yang et al., 2013). ERK1/2 regulates cell survival, differentiation, and proliferation. The involvement of JNK and p38MAPK in inflammation is characterized by their ability to impede cell cycle progression and facilitate apoptosis. Moreover, these signaling pathways may exert crucial pro-apoptotic functions in damaged intestinal epithelial cells (He et al., 2015). Luo et al. (2023) demonstrated that LPS treatment markedly upregulated the mRNA expression of p38 MAPK in the ileum of mice. Upon activation, the p38 MAPK signaling pathway induces robust oxidative stress response and triggers the release of a plethora of inflammatory factors, including TNF-α, IL-1β, and IL-6. Consequently, this leads to an increase in crypt depth and a decrease in villus height and the ratio of villus height to crypt depth (V/C) of the intestine. Simultaneously, there was a significant reduction observed in the expression levels of intestinal barrier proteins such as zonula occludens 1 (ZO-1), occludin, claudin, mucin 2 (MUC2), and junctional adhesion molecule 2 (JAM2). The activation of the MAPK pathway has been demonstrated to be closely associated with the impairment of intestinal barrier function, which is attributed to the phosphorylation of myosin light chain (MLC) (Raj et al., 2023). Phosphorylation of MLC by myosin light chain kinase (MLCK) regulates cellular actomyosin contraction, a crucial step in maintaining barrier integrity through the opening of paracellular pathways (Turner et al., 1997). After MAPK activation, the phosphorylation of MLCK is increased by a significant number of inflammatory factors (such as TNF-α), subsequently leading to MLC phosphorylation and ultimately resulting in the disruption of intestinal tight junctions and an increase in intestinal permeability. Moreover, inhibition of MLCK enhances the barrier function of TNF-α-stimulated intestinal epithelial cells (Zolotarevsky et al., 2002). In addition, TNF-α can also exert an inhibitory effect on the promoter activity of occludin, leading to the rearrangement of ZO-1 and claudin-1 (Wang et al., 2005). Wang et al. (2017) demonstrated that IL-1β induces phosphorylation of p38 MAPK, upregulates MLCK expression, and enhances paracellular permeability in the intestinal epithelial cells. Additionally, they discovered that curcumin inhibits IL-1β-induced activation of p38 MAPK, thereby attenuating the increased expression of MLCK and safeguarding the integrity of the intestinal epithelial barrier against translocation of bacterial LPS from the intestine into systemic circulation. It has been reported that the JNK inhibitor SP600125 has been shown to reduce intestinal inflammatory response and prevent intestinal barrier breakdown by increasing ZO-1 and closure protein expression (Samak et al., 2015). Similarly, Sotetsuflavone exhibited protective effects on the intestinal barrier by suppressing the JNK and p38 signaling pathways in inflammatory conditions (Ge et al., 2023). Xu et al. (2024b) demonstrated that through the inhibition of inflammatory macrophages, regulation of extracellular redox homeostasis, and downregulation of the MAPK/ERK signaling pathway, it is possible to suppress over-activated inflammation and restore cell-tight junction proteins. This leads to a reshaping of the intestinal microenvironment and achieves the purpose of treating endotoxemia.

### 4.2 ApoM/S1P signaling pathway

**FIGURE 3:**
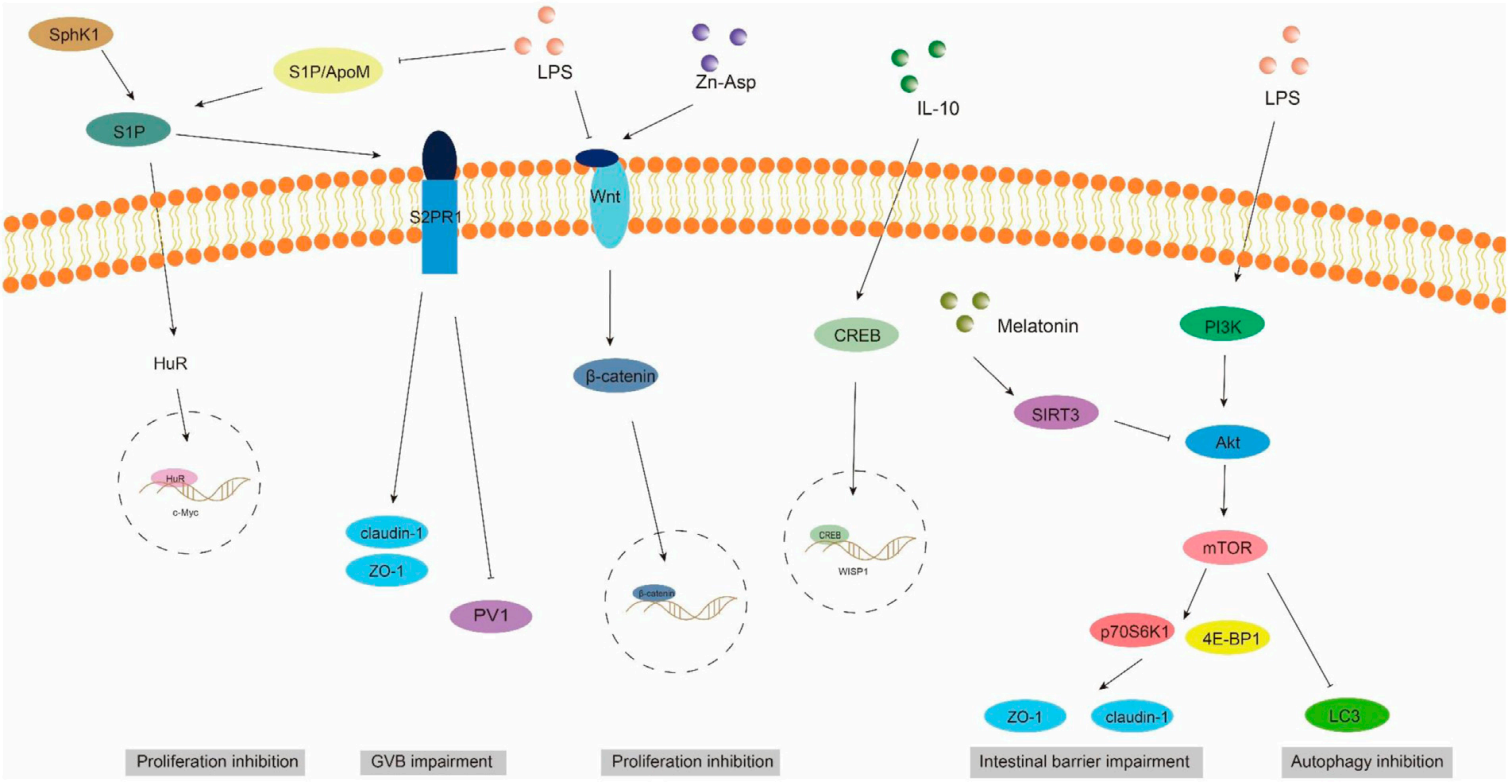
ApoM/S1P, Wnt/beta-catenin, and PI3K/AKT/mTOR signaling pathways involved in intestinal barrier damage. S1P, sphingosine 1-phosphate; S2PR1, sphingosine 2-phosphate receptor 1; SPHK1, sphingosine kinase 1; PV1, plasmalemmal vesicle-associated protein-1.

Gut vascular barrier (GVB), a novel anatomical structure in the mouse and human gut described by Spadoni et al., in 2015, is composed of vascular endothelial cells, pericellular fibroblasts, enteric glial cells, as well as junction complexes including tight junctions (TJs) and adhesion junctions (AJs) (Ge et al., 2020). GVB serves to prevent the entry of microorganisms into the bloodstream and regulate antigen translocation (Sorribas et al., 2019). As an intestinal barrier, GVB has gained increasing attention in recent years. Studies have found that GVB may be impaired in sepsis and its permeability increases (Liu et al., 2020). Sphingosine-1-phosphate (S1P) is a sphingomyelin metabolite that exhibits diverse biological activities, including involvement in cell growth, apoptosis, and regulation of the immune and clotting systems through binding to G protein-coupled receptors. Within the intestinal epithelium, S1P serves as a crucial regulator of epithelial cell barrier function by activating the receptor S1PR1 located in intestinal blood vessels. This activation leads to an increase in trans-monolayer electric resistance of endothelial cells and helps maintain vascular integrity during inflammatory bowel disease (Karuppuchamy et al., 2017). The primary carrier of S1P in plasma is ApoM, with approximately 5% of HDL particles containing ApoM (Frej et al., 2016). In conclusion, the ApoM/S1P axis plays a crucial role in safeguarding the integrity of the intestinal barrier (Li et al., 2023a). Studies have demonstrated that LPS, TNF-α, and IL-1 exert inhibitory effects on hepatic ApoM production (Jiang et al., 2015). Kumaraswamy et al. (2012) observed a significant reduction in plasma ApoM levels among patients with varying degrees of sepsis, septic shock, and SIRS compared to healthy controls, with the extent of reduction reflecting the severity of SIRS/sepsis. Christensen et al. (2016) confirmed an increased intestinal permeability in ApoM (−/−) mice as opposed to ApoM (+/+) mice, suggesting that disruption of the ApoM/S1P axis is responsible for heightened intestinal leakage and damage to the gut-vascular barrier. Sphingosine kinase (SPHK) is a rate-limiting enzyme involved in the synthesis of S1P and plays a pivotal role in maintaining the integrity of the intestinal epithelial barrier (Li et al., 2023d). Chen et al. (2023) discovered an elevation in plasma lipopolysaccharide (LPS) levels in mice with alcoholic liver disease, accompanied by a reduction in SPHK2 protein expression and its regulated S1P, ultimately leading to disruption of the intestinal barrier. Their study substantiated that targeting SPHK2 and increasing S1P levels can ameliorate gut microbiota, reduce plasma LPS levels, and restore intestinal barrier function. In addition, Li et al. (2020) discovered that berberine can augment plasma ApoM and APOM-binding S1P levels during sepsis by inhibiting gluconeogenesis, insulin resistance, and secretion of pro-inflammatory molecules. Subsequent investigations revealed that APOM-bound S1P reduced the expression of PV1, an endothelial permeability marker induced by LPS. Furthermore, the levels of occludin and β-catenin suppressed by LPS were elevated (Li et al., 2020; Spadoni et al., 2015). It is postulated that targeting the ApoM/S1P axis could be a novel approach for treating LPS-induced intestinal barrier damage (Figure 3).

### 4.3 Wnt/beta-catenin signaling pathway

The WNT pathway is a complex signaling network that encompasses the classical WNT/β-catenin pathway and the non-classical WNT pathway. It plays a pivotal role in various biological processes, including cell proliferation, apoptosis, and migration (Moparthi and Koch, 2019). The translocation of β-catenin to the nucleus occurs following activation by the Wnt ligand, thereby facilitating transcription of downstream gene transcription factors that play a pivotal role in shaping the morphology, growth, and regeneration of intestinal epithelial cells (Jung and Park, 2020). Within the gastrointestinal tract, activation of the Wnt/β-catenin signaling pathway is indispensable for maintaining epithelial homeostasis and holds dominance in recognizing and sustaining epithelial stem cells (Kuhnert et al., 2004) (Figure 2). Moreover, this pathway exhibits close association with inflammatory signaling pathways such as NF-κB and MAPK signaling, exerting influence on epithelial homeostasis and tissue regeneration; inhibition of this pathway results in loss of intestinal crypts and tissue denaturation (Moparthi and Koch, 2019). Xie et al. (2020) reported an over-activation of the Wnt/β-catenin signaling pathway following intestinal infection, resulting in aberrant proliferation of intestinal stem cells and crypts, leading to the disruption of the intestinal mucosal barrier and subsequent onset of diarrhea. Mouries et al. (2019) confirmed that fatty liver disease development is accompanied by impairment of GVB and the intestinal barrier, which is closely associated with perturbations in the WNT/β-catenin signaling pathway. Additionally, driving β-catenin activation in endothelial cells prevents damage to GVB and inhibits NASH progression. Studies have demonstrated that during the onset of weaning stress, piglets experience a range of complications including impairment to the integrity of the intestinal epithelial barrier, transient intestinal inflammation, and diarrhea (Wang et al., 2024), which are closely associated with perturbations in the wnt/β-catenin signaling pathway (Wang et al., 2022). It has been reported that upregulation of β-catenin expression can impede the proliferation of pathogenic bacteria, thereby mitigating weaning stress-induced intestinal inflammation and damage to the intestinal barrier (Tong et al., 2023). In addition, the repair of the intestinal mucosal barrier by the anti-inflammatory factor IL-10 has been demonstrated to partially rely on the activation of the Wnt pathway in epithelial cells (Quiros et al., 2017). Regulating the Wnt/β-catenin pathway can also confer protection on GVB function in sepsis (He et al., 2018). The findings of Zhou et al. (2020) demonstrated that zinc L-aspartate (Zn-Asp) effectively augmented the renewal and regeneration of intestinal stem cells (ISCs) through activation of the Wnt/β-catenin signaling pathway. Additionally, it successfully mitigated inflammation in jejunal epithelial cells and preserved intestinal barrier integrity against deoxynivalenol (DON)-induced damage. The preservation of intestinal stem cells (ISCs) is crucial for the sustained regeneration and repair of the intestinal mucosal epithelium following injury, as ISCs possess the capacity to generate multiple cell lineages within the intestinal epithelium, which relies on the proper functioning of the Wnt/beta-catenin signaling pathway (Ma et al., 2023).

### 4.4 PI3K/AKT/mTOR signaling pathway

Autophagy exerts beneficial effects on cellular, tissue, and organ homeostasis, while also playing a crucial role in maintaining intestinal barrier function (Wu et al., 2019). The phosphatidylinositol 3-kinase (PI3K)/Protein kinase B (AKT)/Mammalian target of rapamycin (mTOR) signaling pathway serves as a pivotal transduction factor in autophagy and is involved in the regulation of diverse cellular functions such as cell survival, growth, proliferation, and metabolis (Guo et al., 2019). It has been documented that LPS induces a significant upregulation of mRNA and phosphorylation levels within the AKT/PI3K/mTOR signaling pathway in mice, leading to intestinal inflammatory response and impairment of barrier function. Simultaneously, inhibition of the AKT/PI3K/mTOR signaling pathway can effectively safeguard the intestine against LPS-induced damage to its barrier integrity (Cheng et al., 2024). Wang et al. (2024) demonstrated that LPS significantly attenuated autophagosome formation and suppressed the expression of LC3, a key protein involved in autophagy, within the gastrointestinal tract. Consequently, this impaired elimination of damaged cellular components, leading to heightened intestinal oxidative stress and an exaggerated immune response. Subsequently, they discovered that inhibition of the PI3K/Akt/mTOR signaling pathway reversed LPS-induced suppression of autophagy, thereby mitigating damage to the intestinal barrier. The study conducted by Xu et al. (2021) showed that melatonin induces the upregulation of Sirtuins3 (SIRT3), which in turn modulates the AMPK/mTOR pathway and enhances autophagy, thereby mitigating small intestine damage in sepsis. In addition to affecting autophagy, the activation of the PI3K/Akt/mTOR signaling pathway in intestinal epithelial cells is also closely associated with the expression of tight junction proteins (Wang et al., 2015). Akt activation facilitates mTOR phosphorylation, leading to downstream substrate activation including p70S6K1 and 4E-BP1, which subsequently promote protein synthesis (Manning and Cantley, 2007). Yan and Ajuwon (2017) discovered that stimulation with LPS results in a reduction in the levels of phosphorylated Akt and total Akt, subsequently leading to a decrease in the abundance of phosphorylated 4E-BP1. This cascade effect ultimately causes a decline in the expression of tight junction proteins such as occludin, claudin-4, ZO1and 2. Furthermore, they observed that upregulation of Akt signaling counteracts the LPS-induced decrease in tight junction protein synthesis. These findings suggest that disruption of the epithelial barrier induced by LPS may be achieved through inhibition of the Akt/mTOR signaling pathway. It can be speculated that the PI3K/Akt/mTOR signaling pathway may lead to LPS-induced intestinal barrier damage through autophagy and regulatory protein synthesis. Inhibition of PI3K/Akt/mTOR may enhance autophagy while potentially reducing protein synthesis, thus emphasizing the criticality of maintaining a balanced state within the PI3K/Akt/mTOR signaling pathway to safeguard intestinal barrier integrity (Figure 3).

## 5 Effects of microgravity on intestinal barrier

**FIGURE 4:**
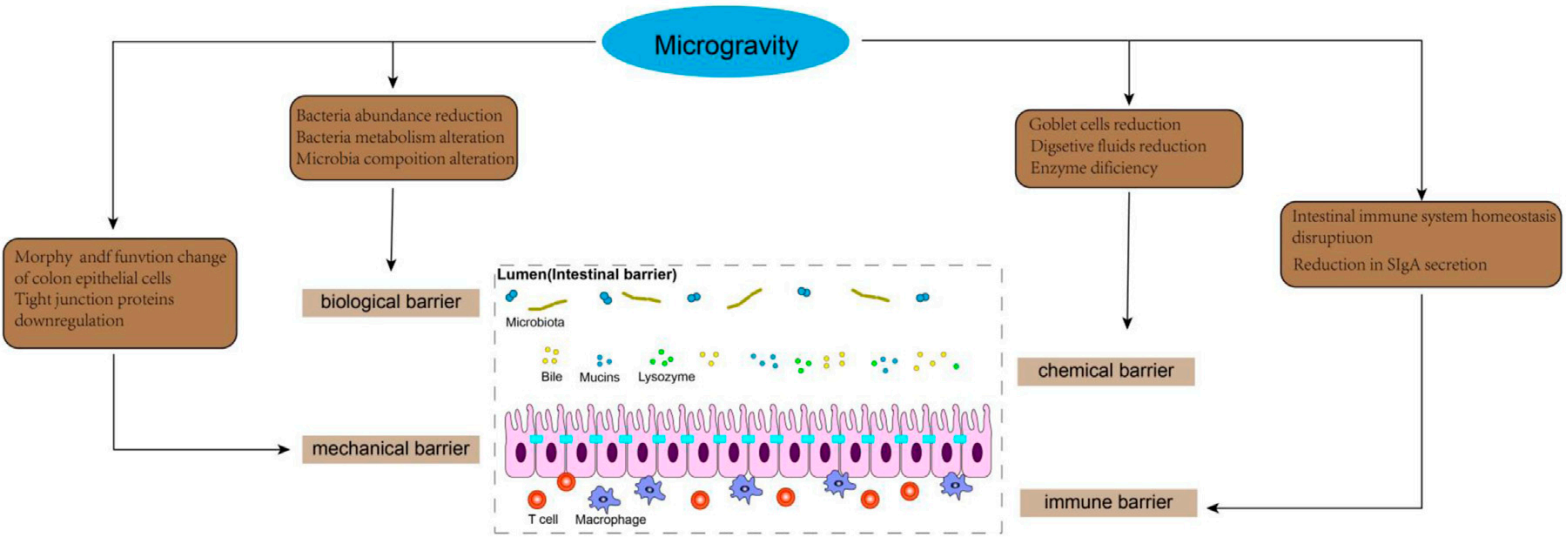
Effects of microgravity on intestinal barrier.

Microgravity is the predominant characteristic of the space environment, which can induce physiological adaptation and pathophysiological alterations in multiple systems, such as muscle atrophy, bone demineralization, and immune system dysregulation (Nie et al., 2024). As a vital interface with the external milieu, the intestinal barrier function plays a crucial role in safeguarding the internal homeostasis by effectively impeding the entry of harmful substances. In recent years, increasing attention has been devoted to investigating the impact of microgravity on gastrointestinal physiology and function, particularly pertaining to the intestine (Figure 4). Moreover, despite its close interconnection with other extraintestinal organs, our understanding of gut-organ axis under microgravity conditions remains limited. Future comprehensive exploration into the intricate interactions between gut and extraintestinal organs within a microgravity environment will significantly contribute to unraveling the mechanisms underlying intestinal barrier function in space.

### 5.1 Intestinal mechanical barrier

The intestinal mechanical barrier is primarily comprised of intestinal epithelial cells (IEC), intercellular junctions, basement membrane, and the submucosal lamina propria, which collectively form the structural foundation of the intestinal mucosal barrier. IEC mainly consists of absorptive enterocytes, goblet cells and intestinal endocrine cells (He et al., 2021). The intercellular junctions in intestinal epithelial cells consist of tight junctions, adherens junctions, gap junctions, and desmosomes. The tight junction complex in the intestinal epithelium, comprising ZO-1, β-catenin, cadherin, claudins, and occludin proteins, plays a crucial role in regulating intestinal permeability. The intact structure of intercellular junctions effectively seals the gap between adjacent epithelial cells, thereby impeding the infiltration of bacteria and toxins into the lamina propria of the intestinal mucosa (Crowley et al., 2024). The occurrence of various adaptive and pathophysiological changes in the structure and physiology of the digestive tract has been reported in a microgravity environment, including the disruption of intestinal microvilli architecture and a significant decrease in microvilli surface area (Li et al., 2015). Spaceflight lasting from 7 to 18 days may compromise the morphology and functionality of colon epithelial cells (Rabot et al., 2000). Furthermore, compared to rats exposed to normal gravity, tail-suspended rats exhibited a significant increase in apoptosis of IECs in the ileum, which was associated with upregulated expression of pro-apoptotic protein Bax and downregulated expression of anti-apoptotic protein Bcl-2 (Jin et al., 2018). As the primary proteins in tight junctions, claudin-1 and claudin-5 play a pivotal role in maintaining the integrity of the epithelial barrier (Abdulqadir et al., 2023). Additionally, E-cadherin serves as a fundamental constituent of adhesion junctions, ensuring mechanical strength and stability to the intestinal lining (The zonula adherens matura redefines the apical junction of intestinal epithelia). Li et al. (2021) discovered that tail suspension led to a decrease in the expression of occludin and zonula occludens-1 (ZO-1), while increasing the concentration of DAO and D-lactic acid in plasma. These findings suggest that simulated weightlessness may impair the intestinal barrier function by disrupting tight junctions and enhancing intestinal permeability. Similarly, Jin et al. (2018) demonstrated a significant reduction in the expression of tight junction proteins such as occludin, claudin-1, claudin-5, and E-cadherin in the ileum of rats after hindlimb unloading for 21 days. The study also demonstrated that under simulated microgravity conditions, the downregulation of tight junction expression was closely associated with the activation of TLR4/MyD88/NF-κB signaling. IECs cultured in simulated microgravity using a rotating wall vessel (RWV) for 18 days prior to seeding on normal gravity condition exhibited reduced junctional ZO-1 localization and FITC-dextran (FD4) permeability, compared with static cells (Alvarez et al., 2019). This study suggests that simulated microgravity can induce a potential and sustained susceptibility to epithelial barrier disruption after being removed from the microgravity environment. The study conducted by Wang et al. (2021) demonstrated that exposure to simulated microgravity for a duration of 3 weeks resulted in impairment of the intestinal mucosal barrier, characterized by reduced goblet cell count, enlarged intercellular space, downregulated adhesion molecules, and increased intestinal permeability in rats. Subsequently, employing proteomics techniques, they discovered that simulated microgravity significantly suppressed the expression of adhesion molecules and disrupted several signaling pathways associated with metabolism, adhesion plaques, and regulation of actin cytoskeleton. Notably, Wang et al.'s findings showed that the downregulation of adhesion molecules and the upregulation of myosin-regulated light chain (MLC) phosphorylation mediated by myosin light chain kinase (MLCK) contributed to intestinal barrier dysfunction during simulated microgravity injury (Wang et al., 2021). It can be speculated that the regulation of epithelial MLCK could potentially offer a novel approach for addressing intestinal barrier injury in microgravity. Additionally, intestinal interstitial connective tissue plays a crucial role in maintaining the mechanical barrier function and osmotic balance of the intestines. Research has demonstrated that specific structural and functional rearrangements of the intestinal connective tissue occur in microgravity conditions (Shishkina et al., 2024). Shishkina et al. (2024) found that the content of fibrous extracellular matrix within the connective tissue in the intestinal wall of C57BL/6N mice after 30 days of spaceflight was significantly reduced, especially the expression of reticular skeleton in the lamina propria and the muscularis externa. This is intricately associated with matrix metalloproteinases (encompassing mast cell proteases) that actively contribute to the gravity-induced adaptations in the intestines (Shishkina et al., 2024; Atiakshin et al., 2023a). In summary, the impact of microgravity on the intestinal epithelium, intercellular connections, and connective tissue of the intestinal wall leads to a profound alteration in the mechanical barrier function of the intestine.

### 5.2 Intestinal chemical barrier

The chemical barrier primarily consists of mucus secreted by the intestinal epithelium, digestive fluid, and antibacterial substances released by probiotics. Mucin (MUC2), which is predominantly produced by intestinal goblet cells and epithelial cells, serves as the principal constituent of the mucous layer covering the intestinal epithelium surface. Structurally resembling bacterial adhesion receptors, MUC2 hinders bacterial attachment to intestinal epithelial cells through competitive binding sites, thereby facilitating bacteria retention within the mucosal layer and subsequent expulsion during intestinal peristalsis (Cheong et al., 2024). It has been reported that the expression of mucin and the number of goblet cells in the gut of Sprague-Dawley rats were found to be reduced after 14 days of space flight, when compared to age-matched ground-based controls (Rabot et al., 2000). Similarly, a decrease in MUC-19 expression was observed in the digestive acinar cells of mice flown on the US space shuttle Atlantis (STS-135). The researchers also suggest that identifying changes in salivary mucin may facilitate the development of non-invasive methods for assessing astronauts’ digestive physiological state (Dagdeviren et al., 2018). Digestive juices play a crucial role in safeguarding the integrity of the gastrointestinal tract. The presence of digestive fluid within the intestines serves to dilute toxins, while also facilitating the removal of pathogenic bacteria from adhering to the intestinal epithelium (Wang et al., 2019). Previous studies indicated an initial increase in intestinal and bile secretions among volunteers subjected to bed rest for a duration of 2 months. However, it was observed that the secretion of digestive fluids gradually declined after this period, potentially contributing to prolonged simulated microgravity-induced injury to the intestinal barrier (Hargens and Vico, 1985). Digestive enzymes are crucial for maintaining normal intestinal function. Studies have shown that Mongolian gerbils subjected to a 12-day space flight exhibited significantly reduced trypsin levels in the stomach and jejunal walls. This enzymatic deficiency led to inadequate mitosis of smooth myocytes in the intestinal walls, resulting in thinning of the smooth muscle layer and subsequently affecting gastrointestinal motility (Atiakshin et al., 2023b). Although direct examination of the impact of digestive enzymes on gut barrier function was not conducted, the correlation between these two factors was evident (Zheng et al., 2024).

### 5.3 Intestinal biological barrier

The biological barrier is a microecosystem established by the symbiotic bacteria in the intestinal cavity in a specific proportion. Intestinal microbiota plays a crucial role in regulating the integrity of the intestinal barrier and host wellbeing, and maintaining a healthy gut environment necessitates stability in terms of species composition, abundance, and localization. They not only adhere closely to the surface of intestinal epithelial mucosa to form a bacterial membrane barrier but also enhance tight junction protein proliferation, promote secretion of the intestinal mucus layer and IgA, as well as interact with other components of the intestinal barriers (Ge et al., 2020). It has been reported that simulated microgravity significantly reduces the abundance of bacteria associated with anti-inflammatory effects, such as Subdoligranulum, Faecalibacterium, Fusicatenibacter, Butyricicoccus, and Lachnospiraceae-NK4A136-0 group when compared to normal gravity (Han et al., 2022). Additionally, KEGG pathway analysis unveiled that microgravity exerts significant impacts on the metabolism of gut microbiota, including pyrimidine, fatty acid, glyoxylate and dicarboxylate, peptidoglycan biosynthesis, as well as carbon fixation in photosynthetic organisms (Siddiqui et al., 2022). The microgravity environment may disrupt the human intestinal microbiota and subsequently compromise the integrity of the intestinal biological barrier. Jin et al. (2018) demonstrated that hindlimb unloading (HU) resulted in a reduction in the abundance of *Clostridium*, a butyric acid-producing bacterium. Butyric acid plays a crucial role as a regulatory factor in the proliferation and differentiation of intestinal epithelial cells. The decrease in butyric acid content may compromise the morphology of intestinal epithelial cells, thereby impairing intestinal barrier function and increasing intestinal permeability (Koh et al., 2016). Many digestive disorders such as diarrhea, intestinal stress, and ulcerative colitis have been closely associated with alterations in butyric acid levels (Koh et al., 2016). Interestingly, studies have revealed that 9 days of space flight led to a significant increase in short-chain fatty acids (SCFAs) concentration within rat cecal contents; however, there was a concurrent decrease observed specifically for butyric acid proportion (Rabot et al., 2000). Shi et al. (2017) discovered that, in comparison to the ground control group, HU resulted in a significant alteration of the intestinal microbiota composition characterized by an expansion in Firmicutes and a reduction in Bacteroidetes. This dysbiosis of gut flora led to a decline in intestinal goblet cell count, epithelial cell turnover rate, as well as the expression of genes associated with defense mechanisms and inflammatory responses among HU mice. Subsequent investigations demonstrated that alterations in gut microbiota increased susceptibility to colitis development in HU mice. Notably, transplantation of fecal matter from normal mice into HU mice ameliorated the damage inflicted upon the intestinal barrier. Probiotics can effectively address gastrointestinal issues that arise during spaceflight and enhance the function of the intestinal barrier by competing with pathogens, reinforcing tight junctions between intestinal epithelial cells, producing vital metabolites, and interacting with host cells to promote physiological and immune alterations (Cunningham et al., 2021). Shao et al. (2017) demonstrated that L. acidophilus probiotics exhibit resilience in stressful microgravity conditions and persist for an extended period within the gastrointestinal tract while maintaining their adhesion ability, thereby preserving the integrity of the intestinal epithelial barrier and preventing pathogen infiltration. The stability of the Freeze-dried *Lactobacillus* casei Strain Shirota capsule was assessed during a 1-month period aboard the International Space Station, revealing its potential to enhance innate immunity and restore gut microbiome equilibrium (Sakai et al., 2018). The value of probiotics in the space environment is gradually being unraveled (Bharindwal et al., 2023). It is imperative to further investigate the alterations of probiotics in the microgravity environment and apply them to address the issue of intestinal biological barrier breakdown that occurs during spaceflight.

### 5.4 Intestinal immune barrier

The intestinal immune barrier consists of gut-associated lymphoid tissue (GALT), diffuse immune cells and secretory immunoglobulin (SIgA). GALT primarily consists of mesenteric lymph nodes (MLN) and lamina propria lymphocytes. GALT plays a crucial role in maintaining the stability of the intestinal immune environment by timely eliminating danger signals. In response to innocuous stimuli, GALT can activate the mechanism of immune tolerance, thereby ensuring the body remains under low feedback immune surveillance (Cukrowska et al., 2017). SIgA is secreted by intestinal immune tissue and plays a crucial role in humoral immunity. It has ability to specifically bind to bacterial antigens, thereby inhibiting bacterial adhesion. Its deficiency can significantly increase the risk of intestinal fistulas and bacterial translocations (Wang et al., 2019). Additionally, macrophages and natural killer cells present in the lamina propria of the intestinal mucosa serve as vital constituents of the intestinal immune barrier and actively participate in immune responses related to enteric functions (Ge et al., 2020). The study conducted by Li et al. (2015) demonstrated that, in comparison to control mice, the population of Treg cells and IL-10 levels in the gut of HU mice were reduced by more than two-fold, while neutrophils and IL-1β exhibited an approximately two-fold increase. These findings provide confirmation that disrupted intestinal immune system homeostasis in mice exposed to simulated microgravity results in a pro-inflammatory shift within the intestinal microenvironment and heightened susceptibility to colitis. The disturbance of the intestinal immune system during space flight is closely associated with alterations in the gut microbiota. Jin et al. (2018) reported that alterations in the gut microbiota of HU rats, characterized by an expansion of *Bacteroides* and a reduction in firmicutes, resulted in significant production of enterogenic endotoxin. This subsequently activated the TLR4/MyD88/NF-κB signaling pathway, leading to increased levels of pro-inflammatory cytokines and decreased SIgA levels, ultimately disrupting intestinal immune homeostasis. Additionally, research has demonstrated that in simulated microgravity environments, the composition of the intestinal microbiota becomes imbalanced, characterized by an elevated proportion of anaerobic and biofilm-forming bacteria, while the proportion of aerobic and Gram-negative bacteria decreases. Moreover, bile acid metabolism is disrupted under conditions of weightlessness (resulting in decreased levels of glycine ursodeoxycholic acid, glycine chenodeoxycholic acid, glycine deoxycholic acid, and glycine cholic acid). Collectively, these factors contribute to a significant rise in intestinal oxidative stress and inflammatory markers in HU rats leading to a reduction in SIgA secretion (Wang et al., 2024). Currently, there is a limited body of research on the intestinal immune barrier in a microgravity environment. Further investigation into the interplay between the immune barrier and other barriers under weightlessness is crucial for comprehending alterations in intestinal function during microgravity conditions.

## 6 The intervention strategies to intestinal barrier damage

### 6.1 Nutritional support

Appropriate nutritional support can promote the repair and regeneration of intestinal mucosa and enhance the defense function of intestinal barrier. Leman Arslan Ariturk et al.’s studies have shown that Docosahexaenoic acid (DHA) can reduce the production of reactive oxygen species, reduce the level of pro-inflammatory cytokines, prevent neutrophil infiltration, etc. Thereby reducing epithelial shedding of the colon and improving glandular structure and mucosal integrity (Ariturk et al., 2024). Yin et al. found that dietary fiber from sweetpotato residues (SRDF) can significantly improve intestinal barrier function by improving intestinal morphology and permeability and inhibiting inflammatory response (Yin et al., 2024). Li et al. (2024) demonstrated that VD/VDR can promote Notch-1 transcription to maintain intestinal tight junction integrity and barrier function. Lu et al. (2024) showed that dietary α-Ketoglutarate (AKG) can prevent mitochondrial dynamic dysfunction, endoplasmic reticulum stress, and mitochondria-associated endoplasmic reticulum membrane disorder, ultimately alleviating LPS-induced intestinal damage. Therefore, through reasonable selection of nutritional support methods, optimization of nutritional formula, attention to the detailed management of nutritional support, avoidance of intestinal damage factors and formulation of personalized nutritional support programs, we can effectively protect the intestinal barrier function and promote the recovery of patients.

### 6.2 Drug treatment

Antibiotics usually rapidly sterilize most bacteria. More and more evidence shows that antibiotics can effectively intervene in intestinal barrier damage, such as rifaximin can significantly increase the level of serum long chain fatty acids and carbohydrate metabolic intermediates, and then affect serum pro-inflammatory cytokines and secondary bile acids, thereby improving the structure of intestinal microbiota and intestinal immune function (Bajaj, 2016). Due to the limitation that antibiotics cannot specifically change the ecology of intestinal flora, bacterial therapy has gradually emerged because of its unique advantages. Probiotics can regulate blood metabolites related to intestinal microbiota, such as cytokines, amino acids and vitamins, which have an impact on intestinal microbiota and thus intervene in intestinal damage (Liu et al., 2021). Studies have shown that probiotics can colonize the human gut and improve the balance of intestinal microbiota. It can improve the integrity of intestinal barrier and reduce intestinal damage by alleviating oxidative stress, enhancing immune response, and increasing the production of short-chain fatty acids (Li et al., 2024; Toritsuka et al., 2024). In addition, other drug interventions can also effectively deal with intestinal barrier damage. Fan et al. (2024) research has shown that Methane saline (MS) can reduce iron death by regulating Nrf2/HO-1 signaling pathway and reduce intestinal ischemia-reperfusion damage. Liu et al. (2024) found that p-Hydroxybenzaldehyde (HD) can combat oxidative stress through the Keap/Nrf2/HO-1 pathway and NF-κB/AP-1 pathway to prevent intestinal barrier damage. Nowadays, many Chinese herbal decoction have shown good therapeutic effect on intestinal barrier damage. For example, modified Zhenwu Decoction can improve intestinal barrier function of experimental colitis by activating sGC mediated cGMP/PKG signaling (Xu et al., 2024c); Paeoniae decoction (PD) can be regulated by intestinal flora and ILC3 interaction to repair chronic colitis intestinal mucosa damage (Huang et al., 2024); Sijunzi decoction can reduce intestinal epithelial barrier damage by regulating intestinal flora and improving inflammation (Li et al., 2024). In a word, with the continuous deepening of traditional Chinese medicine research and the continuous development of modern science and technology, traditional Chinese medicine decoction has broad application prospects and important research value in the treatment of intestinal barrier damage. It is believed that traditional Chinese medicine decoction will play a more important role in the treatment of intestinal barrier damage.

### 6.3 Other interventions

Chen et al. found that moxibustion can improve intestinal barrier function by regulating blood lipids, improving insulin resistance, and alleviating inflammation (Chen et al., 2024). Studies by Liu et al. have shown that Electroacupuncture (EA) can regulate the expression of Corticotropin-Releasing Factor (CRF) and its receptor in the brain-gut interaction pathway through the CRF signaling pathway, thereby reducing inflammatory response and damage to the intestinal mucosal barrier (Liu et al., 2024). Sun et al. used autoinducer-2 to enhance the expression of tight inducer protein to reduce intestinal damage (Sun et al., 2024), maintain water and electrolyte balance, reduce intestinal peristalsis and other measures also help improve intestinal barrier damage. In addition, for severe intestinal barrier damage, surgical treatment may be required, such as removal of dead tissue and reconstruction of the intestine.

In conclusion, a multi-faceted strategy is needed to mitigate intestinal barrier damage. This includes maintaining a balanced diet to support gut health, and the rational use of antibiotics to prevent microbial imbalance. In addition, supplementing with probiotics and prebiotics helps restore the beneficial flora of the gut and enhances the integrity of the gut barrier. Managing stress levels and getting enough rest is also important to prevent damage to the gut barrier. In addition, regular exercise, quitting smoking and limiting alcohol intake are essential for maintaining gut health. Finally, patients who already have intestinal barrier damage need to receive specific medical treatment and to promote recovery and prevent further complications under the close supervision of a medical professional.

## 7 Conclusion

In this review, we initially discuss the role of “gut-organ” axis disruption in the impairment of the intestinal barrier. The compromise of the intestinal barrier can arise from a multitude of factors. Alongside inflammation, stress, tumors, and other intrinsic factors affecting the gastrointestinal tract itself, alterations in extra-intestinal organs also contribute significantly to damage to the intestinal barrier. In general, alterations in the extra-intestinal organs result in damage to the intestinal barrier through a cascade of effects including an inflammatory response, release of metabolites, and influence on intestinal circulation perfusion. Consequently, there is an increase in intestinal permeability and translocation of intestinal flora, leading to the release of enterogenic endotoxins into the bloodstream which act on the original organ via the circulatory system, further exacerbating deterioration of the intestinal organ. The emergence of this detrimental cycle relies on dysregulation within the “gut-organ” axes. It is worth mentioning that in the early stage of certain extra-intestinal organs injury, increased intestinal permeability and the release of endotoxins can be detected, which is necessary for early intervention of the disease. At the same time, the assessment of intestinal barrier damage can also help us to judge the prognosis of extra-intestinal organs. In short, intestinal barrier damage is not only a disease of the intestine itself, but also a “detection agent” for functional changes in other organs. It is noteworthy that during the early stage of certain extra-intestinal organ injuries, there is an increase in intestinal permeability and subsequent release of endotoxins, which plays a crucial role in the timely intervention of the disease. Simultaneously, evaluating the extent of intestinal barrier damage can aid in prognosticating extra-intestinal organ function. In essence, intestinal barrier damage not only affects the intestine itself but also serves as a “detection agent” for functional alterations in other organs.

Secondly, we have summarized the molecular mechanisms underlying intestinal barrier damage (Table 1). In brief, the TLR4 signaling pathway, in conjunction with NF-κB and MAPK, primarily mediates the inflammatory response within the intestine, while inflammatory factors exert a negative impact on tight junction protein expression. The ApoM/S1P pathway predominantly influences the gut vascular barrier (GVB), leading to a significant increase in intestinal permeability. Intestinal stem cells (ISCs) play an indispensable role in sustained regeneration and repair of the intestinal mucosal epithelium, and aberrant activation of WNT/β-catenin can disrupt normal proliferation of ISCs, thereby affecting multiple cell lineages within the intestinal epithelium. Additionally, it is worth noting that LPS-induced intestinal barrier damage may be mediated by autophagy and regulatory protein synthesis through activation of the PI3K/Akt/mTOR signaling pathway. Further exploration into signal pathways associated with intestinal barrier damage holds immense significance for advancing molecular targeted drug development.

**TABLE 1:** Intestinal injury molecular mechanism.

Signaling Pathway	Description	Results	References
LPS/TLR4	Activate the NF-κB and MAPK pathways that regulate inflammation	Impaired intestinal barrier function	Izadparast et al. (2022)
TLR9	NETs induce the activation of TLR9 signaling pathway leading to increased intestinal inflammation	Mechanical barrier disruption	Sun et al. (2021)
TLR4/ATF/CHOP	The activation of the TLR4/ATF/CHOP signaling pathway by ER stress can induce apoptosis or dysfunction in Panzer cells	Lowered antimicrobial peptide secretion, exacerbating injury	Wang et al. (2022a)
TLR	Overexpression of PTX3 can inhibit the TLR signaling pathway, thereby reducing levels of inflammatory factors such as TNF-α, IL-1β, and INF-γ	Promotes ZO-1 and occludin expression, reducing intestinal epithelial damage	Li et al. (2022)
NF-κB	TNF-α and IL-6 activation of TAK1 leads to dissociation of NF-κBp65	The integrity of the intestinal mechanical mucosal barrier is compromised	Cao et al. (2020), Cao et al. (2018)
NF-κB	The activated NF-κB p65 interacts with the promoter region of MLCK and regulates the transcription of MLCK74	The expression of tight junction protein was downregulated and intestinal permeability was increased	Gatica-Andrades et al. (2017)
NF-κB	Affect the expression of MLCK, MLC, ZO-1 and occludin	Affects the integrity and permeability of intestinal epithelial cells	Quan et al. (2020), Zhao et al. (2021)
p38MAPK	Inhibit cell cycle progression, promote apoptosis and Affect the expression of MLCK, MLC, ZO-1 and occludin	Induces oxidative stress responses and triggers the release of inflammatory cytokines, including TNF-α, IL-1β, and IL-6, Affects intestinal barrier function	Luo et al. (2023), Turner et al. (1997), Zolotarevsky et al. (2002), Wang et al. (2005), Wang et al. (2017)
ERK 1/2	Regulates cell survival, differentiation and proliferation	Leads to the remodeling of the intestinal microenvironment	Yang et al. (2013), Xu et al. (2024b)
JNK/SAPK	Inhibit cell cycle progression and promote apoptosis, Affect the expression of ZO-1 and closure protein	Affecting the intestinal barrier	Samak et al. (2015), Ge et al. (2023)
ApoM/S1P	LPS, TNF-α and IL-1 inhibit the production of ApoM, Targeting SPHK2 and increasing S1P levels can improve gut microbiota	Affects intestinal vascular barrier and permeability	Jiang et al. (2015), Christensen et al. (2016)
Wnt/β-catenin	Overactivation of the Wnt/β-catenin signaling pathway leads to abnormal proliferation of intestinal stem cells and crypts	The intestinal mucosal barrier is destroyed	Xie et al. (2020)
Wnt/β-catenin	Driving β-catenin activation in endothelial cells	Prevents damage to GVB and inhibits NASH progression	Mouries et al. (2019), He et al. (2018)
Wnt/β-catenin	Upregulation of β-catenin expression can impede the proliferation of pathogenic bacteria	Reduces intestinal inflammation and intestinal barrier damage	Wang et al. (2022b)
Wnt/β-catenin	Zn-Asp effectively augmented the renewal and regeneration of ISCs through activation of the Wnt/β-catenin signaling pathway	Protects the integrity of the intestinal barrier	Zhou et al. (2020)
AKT/PI3K/mTOR	It is a key transduction factor in the process of autophagy and is involved in the regulation of a variety of cell functions, such as cell survival, growth, proliferation and metabolism	Affects intestinal inflammatory response and barrier function	(Guo et al., 2019) (Cheng et al., 2024)
AKT/PI3K/mTOR	Melatonin induces the upregulation of SIRT3, which in turn modulates the AMPK/mTOR pathway and enhances autophagy	Reduce small intestinal damage	Xu et al. (2021)
AKT/PI3K/mTOR	Through autophagy and regulation of protein synthesis, such as tight junction protein expression	Affects intestinal barrier integrity	Wang et al. (2015), Manning and Cantley (2007)

Finally, we have concluded the alterations in the intestinal barrier under microgravity conditions (Table 2). In a microgravity environment, there are notable changes observed in intestinal epithelial cells and their tight intercellular connections, which contribute significantly to the increased permeability of the intestines. Rresearch has been conducted on the impact of microgravity on the biological barrier of the intestines. It has been discovered that both the composition and metabolites of intestinal microbiota undergo disturbances in a microgravity environment. These alterations in gut flora also influence various aspects such as intestinal epithelial cell formation, immune responses within the intestines, and mucus production. Furthermore, microgravity affects chemical and immune barriers within the intestines by reducing secretion levels of digestive fluids, mucin, and SIgA (Atiakshin et al., 2019). However, limited studies have focused on exploring these effects specifically on chemical and immune barriers under microgravity conditions. Further investigation into how microgravity impacts different aspects of intestinal barriers is crucial for preventing damage to these barriers during space travel while ensuring optimal gastrointestinal health for astronauts. Moreover, this knowledge will provide valuable insights for future space exploration endeavors and potential colonization beyond our planet.

**TABLE 2:** The effects of microgravity on intestinal barrier.

NO	Intestinal barrier	Changes	Reference
1	Mechanical barrier	The disruption of intestinal microvilli architecture and a significant decrease in microvilli surface area	Li et al. (2015)
2	Mechanical barrier	Compromise the morphology and functionality of colon epithelial cells	Rabot et al. (2000)
3	Mechanical barrier	Significant increase in apoptosis in the ileum	Jin et al. (2018)
4	Mechanical barrier	Impair the intestinal barrier function by disrupting tight junctions and enhancing intestinal permeability	Jin et al. (2018) , Abdulqadir et al. (2023), Li et al. (2021), Alvarez et al. (2019)
5	Mechanical barrier	The downregulation of adhesion molecules and the upregulation of myosin-regulated light chain (MLC) phosphorylation mediated by myosin light chain kinase (MLCK)	Wang et al. (2021)
6	Mechanical barrier	Specific structural and functional rearrangements of the intestinal connective tissue	Shishkina et al. (2024), Atiakshin et al. (2023a)
7	Chemical barrier	The expression of mucin and the number of goblet cells in the gut were found to be reduced	Dagdeviren et al. (2018)
8	Chemical barrier	The secretion of digestive fluids gradually declined	Hargens and Vico (1985)
9	Chemical barrier	Significantly reduced trypsin levels in the stomach and jejunal walls	Atiakshin et al. (2023b)
10	Biological barrier	Significantly reduces the abundance of bacteria associated with anti-inflammatory effects	Han et al. (2022)
11	Biological barrier	Significant impacts on the metabolism of gut microbiota	Siddiqui et al. (2022)
12	Biological barrier	A significant alteration of the intestinal microbiota composition	Shi et al. (2017)
13	Immune barrier	Disrupted intestinal immune system homeostasis results in a pro-inflammatory shift within the intestinal microenvironment	Li et al. (2015), Jin et al. (2018)
14	Immune barrier	A significant rise in intestinal oxidative stress and inflammatory markers leading to a reduction in SIgA secretion	Wang et al. (2024d)
